# Mesenchymal stem cells in animal reproduction: sources, uses and scenario

**DOI:** 10.29374/2527-2179.bjvm002524

**Published:** 2024-05-06

**Authors:** Andrei Takeshita de Oliveira, Antonio Rodrigues Ferreira Braga, José Ricardo Fonseca Miranda, Paulo Fantinato-Neto, Carlos Eduardo Ambrósio

**Affiliations:** 1 Undergraduate in Veterinary Medicine, Faculdade de Zootecnia e Engenharia de Alimentos (FZEA), Universidade de São Paulo (USP). Pirassununga, SP, Brazil.; 2 Veterinarian, DSc., Programa de Pós-Graduação em Biociência Animal, FZEA, USP, Pirassununga, SP, Brazil; 3 Veterinarian, DSc., Departamento de Medicina Veterinária, FZEA, USP, Pirassununga, SP, Brazil

**Keywords:** mesenchymal stem-cells, MSCs, animal reproduction, cellular therapy, células-tronco mesenquimais, MSCs, reprodução animal, terapia celular

## Abstract

Studies regarding mesenchymal stem cells turned up in the 1960’s and this cell type created a great number of questions about its functions and applicability in science and medicine. When used with therapeutic intent, these cells present an inclination to migrate to sites of injury, inflammation or disease, where they secrete bioactive factors that stimulates the synthesis of new tissue. In this context, studies using rodents reported that MSCs promoted positive effects in the ovarian function in mice with premature aging of follicular reserve. In female bovines, experimental stem cell-based therapies have been used to either generate new oocytes with *in vitro* quality or stimulate such action *in vivo*. It is also reported, that the intraovarian application of mesenchymal stem cells generates a greater production of embryos *in vitro* and the production of early and expanded blastocysts. Additionally, analysis of ovarian tissue in animal subjected to treatment showed an increase in the number of developing follicles. Nevertheless, the treatments involving stem cells with different modes of application, different sources and different species were able to act on the hormonal, tissue, cellular and metabolic levels, generating positive results in the recovery and improvement of ovarian functions.

## Resumo

Estudos sobre células-tronco mesenquimais surgiram na década de 1960 e esse tipo de célula gerou muitas dúvidas sobre suas funções e aplicabilidade na ciência e na medicina. Quando utilizadas com intuito terapêutico, essas células apresentam tendência a migrar para locais de lesão, inflamação ou doença, onde secretam fatores bioativos que estimulam a síntese de novos tecidos. Nesse contexto, estudos utilizando roedores relataram que as CTM promoveram efeitos positivos na função ovariana em camundongos com envelhecimento precoce da reserva folicular. Em fêmeas bovinas, terapias experimentais baseadas em células-tronco têm sido utilizadas para gerar novos oócitos com qualidade *in vitro* ou estimular tal ação *in vivo*. É relatado também que a aplicação intraovariana de células-tronco mesenquimais gera maior produção de embriões in vitro e produção de blastocistos precoces e expandidos. Além disso, a análise do tecido ovariano em animais submetidos ao tratamento mostrou aumento no número de folículos em desenvolvimento. Apesar disso, os tratamentos envolvendo células-tronco com diferentes modos de aplicação, diferentes fontes e diferentes espécies foram capazes de atuar nos níveis hormonal, tecidual, celular e metabólico, gerando resultados positivos na recuperação e melhora das funções ovarianas.

## Introduction

Since the emergence of studies on mesenchymal stem cells (MSCs), which began in 1960 by the Friedenstein group (Friedenstein et al., 1966, 1970) this type of cell gained prominence in the scientific community, being defined as cells capable of extensive self-renewal and of giving rise to at least one type of highly differentiated descendant (Lima & Durli, 2018). Stem cells are widely used in medicine due to their trophic properties (migration, anti-apoptosis, anti-fibrosis, angiogenesis, anti-inflammation), immunomodulation, and protection against oxidative stress.

The mesenchymal stem-cells can be sourced from many tissues, but the most utilized are the adipose tissue, obtained from liposuction or lipectomy (Gimble et al., 2013; Maria et al., 2017; Varghese et al., 2017), neonatal umbilical cord tissue, specifically the conjunctive tissue, Wharton’s jelly and vasculature (Hass et al., 2011; Nagamura-Inoue & He, 2014), and from bone marrow, usually from the pelvic bone and iliac crest for clinical use, and from the femur’s head and neck osteotomy for preclinical research (Pittenger et al., 1999; Lee et al., 2003; Wagey & Short, 2013; Yin et al., 2019).

The tissue regeneration and preservation properties have led stem cells to be used in numerous studies about treatments of diseases related to ligaments, tendons, and bones (Bigham-Sadegh et al., 2012; Broeckx et al., 2019; Kim et al., 2019; Magri et al., 2019; Sharun et al., 2020) and even in issues in the central nervous and cardiovascular system (Ryu et al., 2012; Sarmento et al., 2014; Yang et al., 2021), also extending to diseases related to the animal's physiology, such as diabetes (Zhu et al., 2011).

At the same time, the development of reproductive biotechnologies such as assisted reproductive technologies (ARTs) has enabled a better understanding of animal physiology, driving the development of hormonal regulation protocols (Seneda et al., 2022) and the implementation of techniques such as Fixed-Time Artificial Insemination (FTAI) and Embryo Transfer (ET), which are important in today's livestock industry, mainly for enhancing the efficiency and sustainability of food production worldwide (Butler et al., 2023).

The foundation of cellular treatment with MSCs is related to tissue recovery that stem cells can achieve in the studied regions. In the scenario of animal reproduction, a study conducted in rats demonstrated that the application of MSCs was able to increase the expression of genes related to vascular endothelium growth in ovarian tissue, besides indicating a high concentration of MSCs in the theca and primordial follicle regions (Vural et al., 2019). Another treatment performed with MSCs was able to increase the concentrations of estrogen (E2) and anti-Müllerian hormone (AMH) in rats affected by ovarian insufficiency (Feng et al., 2020; Sen Halicioglu et al., 2022), demonstrating both the hormonal and tissue applicability that cellular treatment can have in animal reproduction.

This review aims to relate the most current findings regarding studies involving mesenchymal stem cells applied to animal reproduction, in order to understand the origin of this group of cells, the methods of collection and cultivation, and the main biological factors that lead to the described alterations in treatments.

## Mesenchymal stem cell

### Definition

The first appearance of what would be called mesenchymal stem cells within the scientific community occurred during the 1960’s to 1970’s by the Friedenstein group. It was observed that a marginal cell subset culture from the bone marrow, morphologically similar to fibroblasts, could be induced to osteogenesis in diffusion chambers in the presence of transitional epithelium (Friedenstein et al., 1966, 1970). These marginal cells subset was defined as adherent colony-forming unit fibroblasts (CFU-Fs) in contrast with non-adherent hematopoietic CFU cells (CFU-Cs) which are granulocyte-macrophage progenitor cell (Jinnai et al., 1984; Nara et al., 1984). The CFU-Fs were initially considered capable of producing cells associated with the skeletal tissue, being able to differentiate *in vitro* not only to osteocytes, but also to chondrocytes, adipocytes and being able to transfer the microenvironment typical of hematopoietic tissues *in vitro* (Friedenstein et al., 1970, 1974). Later, CFU-Fs were observed as feeders for *ex-vivo* culture of hematopoietic stem cells (HSC) (Castro-Malaspina et al., 1980). In 1984, Jinnai, I. was capable of demonstrating that CFU-Fs were positively correlated with erythroid colony-forming cells (CFU-Es) and erythroid burst-forming units (BFU-Es) but not with CFU-Cs.

In the year of 1988, there were papers that permitted a further understanding of the nature of these stem cells, including evidences of circulating factor produced by regenerating marrow that and differentiation of marrow CFU-Fs at distant sites, formation of osteogenic tissue from single-cell suspension of human bone marrow cultured in vivo within diffusion chambers, and the first description of the necessary culture conditions that permitted bone marrow stromal fibroblasts to differentiate in vitro into osteogenic tissue similar to bone (Bab et al., 1988a, 1988b; Maniatopoulos et al., 1988).

Soon, the designation of CFU-Fs evolved to “osteogenic stem cell” or “bone-marrow stromal cells”, terms were adopted to better describe the biological function of the cell subset, claiming evidence that support the existence of a lineage of stromal stem cells within the post-natal organism, present in the soft connective tissue associated with marrow and bone surfaces, capable of giving rise to other cells of the osteogenic lineage and their high ability of self-renewal and multipotentiality (Owen & Friedenstein, 1988; Beresford, 1989).

The acknowledgement of what would be called adult “mesenchymal stem cell” came in 1991 with Caplan et al., who described a small number of cells originated from the embryonic mesoderm capable of division, and their progeny commit to a distinct phenotypic pathway, also reporting that local cuing and the genomic potential interact at each lineage step, controlling the rate and characteristic phenotype of the cells that will compose the emerging tissue (Caplan, 1991).

There are controversies surrounding the term “mesenchymal stem cells” because this term represents a class of mammalian bone marrow and periosteum cells that can be isolated and expanded while maintaining their *in vitro* capability to form bone, cartilage, fat and other tissues when correctly induced (Caplan, 2017). Nevertheless, the name “stem cell” can infer to the general public that these cells will differentiate into regenerative tissue-producing cells, fabricating the missing or diseased tissue, but the fact is that these cells incline to migrate to sites of injury or disease (Silva Meirelles et al., 2009) and secrete bioactive factors that are immunomodulatory and trophic, that are the medicinal factor (Caplan & Dennis, 2006) that will contribute to the patient’s own site-specific and tissue-specific resident stem cells that will commence to construct new tissue as stimulated by bioactive factors secreted by the exogenous MSCs (Le Blanc & Mougiakakos, 2012; Caplan, 2015). Therefore, the MSCs had many names with the same meaning as marrow stromal cells, multipotent stromal cells, mesodermal stem cells and mesenchymal stromal cells, but Caplan, A. I. proposed to call them medicinal signaling cells because of their *in vivo* secretory and primarily at site of injury, disease and inflammation (Caplan, 2017).

### Sources of tissue

There are three main sources of MSCs: bone marrow, adipose tissue and the neonatal tissue like the umbilical cord (Hass et al., 2011). These MSCs supposedly should be confined to a marginal cell population of organs with a perivascular niche, because of the expression in all MSCs of the stroma cell surface marker 1 (Stro-1) and/or α-smooth muscle actin (α-SMA), regardless of the source (Kolf et al., 2007). Because of their fetal nature, umbilical cord MSCs presented better expandability *in vitro* compared to those obtained from adipocyte and bone marrow tissue (Kern et al., 2006). Regardless, the bone marrow source is the most valued since it is better documented and used with more frequency in both preclinical and clinical research (Yin et al., 2019).

### Bone marrow

The bone marrow MSCs (BM-MSCs) usually are isolated from the total marrow obtained from the pelvic bone and iliac crest for clinical use, and from femur’s head and neck osteotomy for preclinical research. This requires an invasive surgical procedure that requires anesthesia and implies nosocomial infection hazards (Pittenger et al., 1999; Lee et al., 2003; Wagey & Short, 2013; Yin et al., 2019). For the isolation of the MSCs from the total bone marrow, a density gradient centrifugation is necessary, with the collection of the mononuclear cell fraction. These mononuclear cells are seeded in culture dishes at low density, about 10^3 to 10^4 cells/cm^2^ (Beyer Nardi & Silva Meirelles, 2006; Sensebé, 2008).

### Adipocyte

Adipocyte MSCs (ASCs) can be isolated from tissue samples obtained after liposuction or lipectomy. This adipose tissue can be visceral or subcutaneous located in the abdomen, brachium, femoral or gluteal areas (Varghese et al., 2017). After that, the fat tissue sample is digested with collagenase followed by red blood cells (RBC) removal with specific RBC lysis followed by a cell filtration. The expansion method is like the BM-MSCs. Today, the ASCs are frequently used because of the less invasive surgical procedures compared to BM-MSCs and natural abundance of MSCs (Gimble et al., 2013; Maria et al., 2017; Varghese et al., 2017).

### Umbilical cord

Mesenchymal stem cells can be isolated from neonatal tissue, especially the umbilical cord. They can be sourced from the whole umbilical cord or its individual compartments: conjunctive tissue, Wharton’s jelly and vasculature (Hass et al., 2011; Nagamura-Inoue & He, 2014). The cell biology procedures for the isolation of MSCs from the umbilical cord may vary depending on the compartment of choice, but it may include an enzymatic digestion of the sample, RBC-specific lysis, cell filtration and density gradient separation. The expansion method is similar to the adult tissue counterparts (Silva Meirelles et al., 2009; Wagey & Short, 2013; Nagamura-Inoue & He, 2014).

### MSCs identity

For the use in preclinical and clinical procedures, a cell heterogeneity is expected from a MSC culture, but it can contain different cells subsets resulting from intrinsic and extrinsic influences like different tissues sources and donors (Sacchetti et al., 2016; Han et al., 2017). Besides multipotent cells, MSC cultures may include diverse but coherent commitment progenitors(Friedenstein et al., 1976). The International Society of Cell Therapy (ISCT) established a criterion in 2006 to define mesenchymal stem cells based in common properties of cultures from different sources: (1) In standard culture conditions, the MSCs must present adherence and a spindle-shape morphology; (2) These cells must show cell surface expression of cluster of differentiation (CD)105, CD73 and CD90, and shouldn’t show expression of CD45, CD34, CD14, CD11α, CD79α, CD19 and HLA-DR antigens; (3) The MSCs shall be able to differentiate into osteoblasts, adipocytes and chondroblasts *in vitro* when correctly stimulated (Dominici et al., 2006).

### MSCs biological functions of clinical interest

The MSCs must have a proliferation function, being able to expand and self-renewal *in vitro*, retaining the fibroblast-like morphology (Cai et al., 2004). They are also able to differentiate into adipocyte, chondroblast and osteoblast *in vitro* within a controlled ambient. This differentiation is perceived morphologically or with biomarkers expressed by each kind of cell (Pittenger et al., 1999). These cells are said to be also capable of supporting the maintenance, expansion and differentiation of HSCs, regulating the homeostasis of the hematopoietic sites *in vivo* (Maitra et al., 2004)*.* They have a pattern of migration within the body: (1) passage or location in a non-specific tissue; (2) homing in native niches; (3) migration towards diseased or damaged tissues (Karp & Leng Teo, 2009).

Mesenchymal stem cells also have a paracrine immunosuppression function regulated by inflammatory factors, that upregulate HLA-class II expressed by some MSCs, suppressing a broad range of immune cells like lymphocytes T and B, natural killers (NK), and affecting the function of myeloid cell such as monocytes, macrophages and dendritic cells (Tse et al., 2003). They regulate both the innate and adaptive immune cells by disrupting their activation, proliferation, maturation, cytokine production, cytolytic production, antibody production (Gao et al., 2016).

## Clinical trials and uses

Mesenchymal stem cells studies and uses in clinical trials had an exponential increase in the last few years (Naji et al., 2019a; Pittenger et al., 2019; Levy et al., 2020; Zhou et al., 2021; Galderisi et al., 2022). The first MSC therapy-based report occurred in 1995, when Hillard Lazarus did a phase I trial with 23 cancer patients receiving intravenous infusion of human bone marrow stem cells. This treatment was done with ex vivo expansion of stromal cells and concluded that the cellular therapy didn´t have shown any adverse reactivity and it was safe to obtain, expand and infuse human bone marrow stromal cells without toxicity (Lazarus et al., 1995). Thus, Lazarus' study was the opening gate for the trials, utilization and its rise in the scenario of mesenchymal cellular therapy.

Until 2023, 1501 studies were found about Mesenchymal stem cells in the Clinical Trials database of the US National Institute of Health. These clinical assays have been done, in great majority, in China, United States and European Union. In this context, about eight to ten studies were still in early phases and even most of them were academia-sponsored - about 60%, - the increase of the industry-sponsored research contributed to the phase's progression (Naji et al., 2019b). From 2011, several MSC products like Cartistem®, HeartiCellgram® and Mesoblast were approved by the Food and Drug Administration (FDA) all over the world (Zhou et al., 2021).

Although these multipotent cells have demonstrated safety and a big promise to be effective to many clinical uses, it had a disorderly growth in clinics offering for the public in the last decade (Rubin, 2018; Zhou et al., 2021). From 2009 to 2014, the number of clinics in the United States (US) providing unproven MSC therapies grew at least 100% yearly and, in 2016, more than 350 US companies were selling stem cells to interventions at 570 clinics. Therefore, even though serious clinical studies have been done with randomized controlled trials, this boom in stem cell clinics selling therapies based on marketing and client testimonials are a threat to the development of the Mesenchymal stem cells therapy (Rubin, 2018; Pittenger et al., 2019).

The main clinical trials in human treatments using mesenchymal cell therapy are to inflammatory and degenerative diseases (multiple sclerosis, Crohn's disease); orthopedics injuries (muscle, bones and cartilages); cardiovascular system; liver, lungs and gastrointestinal tract (cirrhosis, fibrosis); immune rejections (GVHD and transplantations); skin and dermatological; eyes; Diabetes Melitus; Covid-19 and reproductive system (urinary tract, sexual organs and pregnancy conditions) (Wang et al., 2018; Naji et al., 2019a; Guo et al., 2020; Levy et al., 2020; Golchin, 2021; Klerk & Hebrok, 2021; Zhou et al., 2021; Brianna et al., 2022).

In Veterinary Medicine, MSCs have been used as well, whose main sources are bone marrow and adipose tissue (Ivanovska et al., 2022; Prządka et al., 2021). These cellular therapies are mostly applied in horses, dogs and cats in clinical and experimental treatments for joints, ligaments and tendons diseases (Broeckx et al., 2019; Kim et al., 2019; Magri et al., 2019); central nervous system disorders (Meningoencephalitis and spinal cord injuries) (Ryu et al., 2012; Sarmento et al., 2014); muscular diseases (Nitahara-Kasahara et al., 2012; Gibson et al., 2017); bone disorders (Bigham-Sadegh et al., 2012; Sharun et al., 2020); cardiovascular system problems (Yang et al., 2021); gastrointestinal disorders (Pérez-Merino et al., 2015); cutaneous and ocular diseases (Sgrignoli et al., 2019; Enciso et al., 2020; Falcão et al., 2020; Kim et al., 2023) and diabetes (Zhu et al., 2011).

In addition to the clinical and experimental applications for veterinary treatments, animals have been serving like bio models in trials with mesenchymal cell therapies. Although rodents are largely used in trials due to the facility to create, treat and reproduce, suines and dogs are also used and offer advantages, because of the more similar anatomy, physiology and genomic to humans, that permits modeling human diseases (Starkey et al., 2005; Kuzmuk & Schook, 2011). These domestic animal studies permitted the science progress and initiate new possible treatments with regenerative medicine using MSCs.

For fertility in bovine females, since there is a decrease in follicular reserve due to age or any other factors, cell therapy based on stem cells could serve either to generate new oocytes with in vitro quality or to stimulate such action in vivo (Vanni et al., 2017). In this context, studies, mostly done in rodents, reported that MSCs promoted positive effects in the ovarian function in mice with premature aging of follicular reserve (Zhang et al., 2016), in the ovarian reserve itself of female rats via the paracrine route (Li et al., 2017) and in the recovery of fertility and pregnancy capacity of animals that had ovarian lesions induced by chemotherapy treatment (Lima & Durli, 2018).

The effectiveness of these stem cell therapies could be explained by the effects on granulosa cells of the ovarian follicle (Falcão et al., 2020), as well as their trophic properties (migration, anti-apoptosis, anti-fibrosis, angiogenesis, anti-inflammation), of immunomodulation and protection against oxidative stress (Silva Meirelles et al., 2006).

In cow treatments, (Carmo, 2021) reported that the intraovarian application of MSCs is safe and did not lead the animals to inflammatory conditions and (Falcão et al., 2020) demonstrated that intraovarian injection of adult stem cells generated a greater amount of total and viable oocytes compared to untreated ovaries, which resulted in greater production of embryos produced *in vitro*, as well as increased production of early and expanded blastocysts.

## Animal reproduction

An evolution in the use of assisted reproduction techniques has expanded our knowledge of the metabolic and hormonal interactions that structure the estrous cycle of bovine females. Furthermore, ARTs contribute significantly to the efficiency, sustainability, and productivity of livestock (Butler et al., 2023), enabling an increase in the rate of pregnant cows per season and enhancing profitability in livestock farming (Sales et al., 2023). Among the most widely used techniques are artificial insemination (AI), which involves the use of frozen and thawed semen, and embryo transfer (ET), where recipients are prepared to receive embryos fertilized in vivo or in vitro. In addition, hormonal protocols can be employed to synchronize the estrous cycle of animals, allowing for fixed-time artificial insemination (FTAI) and fixed-time embryo transfer (FTET) (Seneda et al., 2022).

### Fixed-time artificial insemination

The estrous cycle bovine consists of two to three waves of follicular growth, where each wave recruits a single dominant follicle. The onset of the follicular wave begins with the release of gonadotropins, resulting in an increase in LH concentration and subsequently FSH, signaling the start of antral follicle development (Hessock et al., 2023). Throughout the cycle, most follicles undergo atresia, and only the dominant follicle reaches the pre-ovulatory stage. A new peak of LH and FSH signals ovulation, marking the beginning of a new wave (Billhaq & Lee, 2023).

In the execution of fixed-time reproduction protocols, hormonal interactions are a key aspect of the technique, comprising three main points: synchronization of the emergence of the follicular growth wave, control of the progesterone phase, and induction of fixed-time ovulation (Seneda et al., 2022; Tschopp et al., 2022).

The reproductive protocol begins with synchronizing the start of a follicular wave. Based on hormonal effects, the combination of progesterone (P4) and estrogen (E2) is widely used in the field (Rocha et al., 2022; Tschopp et al., 2022). In this case, the application of P4 mimics the function of the corpus luteum, preventing an increase in luteinizing hormone concentration, halting the activities of larger follicles. Additionally, the application of E2 prevents smaller follicles from developing, interrupting the rise in follicle-stimulating hormone (FSH) concentration. Thus, the two waves of gonadotropins signaling the start of follicular growth are interrupted; this synchronization is referred to as D0, and after 4 days, the emergence of a new follicular wave can be observed (Oliveira et al., 2018).

After synchronizing the emergence of the follicular wave, the animal enters the progesterone phase, where P4 concentrations remain high. P4-releasing devices are used for hormonal control, aiming to simulate the action of the corpus luteum, maintaining a constant P4 concentration. Around days 5 to 9 of the protocol (D5 to D9), the device is removed, and subsequently, a dose of prostaglandin (PGF2α) is administered. The purpose of PGF2α application, and its analogs, is luteolytic action, occurring within 48 hours after application (Santos Marques et al., 2022).

The final step of the hormonal protocol involves ovulation synchronization, achieved using direct and indirect inductive hormones. Estradiol esters are commonly used indirect inductors in the field; estradiol benzoate (EB) should be applied 24 hours after P4 implant removal, while estradiol cypionate (EC) should be applied on the day of implant removal (Jesus Junior et al., 2023). The difference is related to the half-life of the inductors and the period in which each promotes ovulation effects.

Furthermore, the use of GnRH in ovulation induction for FTAI synchronization protocols aims to improve LH release for the start of the follicular wave, enhance egg quality, and increase conception rates (Rojas Canadas et al., 2023; Sá & Oliveira Júnior, 2023; Velho, 2023). In some cases, there is also the possibility of implementing equine chorionic gonadotropin (eCG) in the protocol; this hormone is used to promote follicular growth. The effect of eCG is based on the stimulation of LH and FSH receptors responsible for follicle development, and its long half-life allows for single-dose usage in the protocol (Fernandes et al., 2021).

### Embryo transfer

The embryo transfer (ET) is a technique where embryos from genetically selected individuals are transferred to a recipient that will carry and give birth to the offspring (Vieira & Pazzim, 2021). This technology allows for an increase in the number of animals derived from selected crosses, enhancing genetic improvement (Berling et al., 2022). The success in ET depends on the presence of a corpus luteum at the time of embryo implantation, ensuring the reproductive physiology continues the gestation process. Thus, the size and quality of the corpus luteum are crucial for ET, and techniques such as transrectal ultrasonography with Doppler are used to evaluate CL perfusion and assess the viability of the transfer (Santos et al., 2023).

Similar to fixed-time artificial insemination (FTAI), hormonal protocols can be implemented in ET to synchronize ovulation, resulting in better control of the reproductive management of embryo recipients. In this case, fixed-time embryo transfer (TETF) uses the same drugs as FTAI for protocol execution (Santos et al., 2023).

Embryos used in ET can be produced in vivo or in vitro. In the case of in vitro embryo production (IVP), several techniques are used to facilitate the process. In this context, ultrasound-guided follicular aspiration (OPU) is the procedure used to collect antral follicles from the animal. Through transrectal palpation, the technician directs a transvaginal probe to the animal's ovary, which is connected to a vacuum system and a needle for aspiration. This allows the collection of follicles, which are subsequently selected for fertilization in the laboratory (Hayden et al., 2022).

It's worth mentioning that the use of equine chorionic gonadotropin (eCG) in protocols for cows subjected to OPU has proven advantageous. eCG increases the quantity of medium and large-sized follicles, enhancing the number of aspirable follicles and improving oocyte viability by reducing polyspermia (Ribas et al., 2018).

### Antral follicular count (AFC’s)

A population of antral follicles in a cow corresponds to the quantity of follicles present in the ovaries throughout the animal's life. The measurement of this parameter is carried out through the count of antral follicles (CFA) larger than 3 mm in diameter, which is performed by transrectal or transvaginal ultrasonography in female cattle (Alward et al., 2023). In this context, animals can be classified into high and low follicle counts.

CFA exhibits high variability within the herd but high repeatability on an individual basis (Morotti et al., 2021) and is related to the genetics of the animal, the functioning of the estrous cycle in females, body condition, and nutrition. This parameter has been linked to the fertility of females based on shorter calving intervals, improved quality and viability of embryos, and enhancements in hormonal interactions related to the estrous cycle and uterine health (Souza et al., 2019).

The Anti-Mullerian Hormone (AMH) is produced by granulosa cells of growing follicles and is responsible for regulating the initial recruitment of primordial follicles to antral follicles. For this reason, it can be used as a tool to estimate the quantity of healthy follicles in an animal (Schwarzmann et al., 2023). Thus, higher concentrations of AMH may be associated with improved fertility in female cattle.

## The application of stem cells in animal reproduction

The varied applications of mesenchymal stem cells in differential treatments are based on the cell's ability to promote tissue recovery. This impact on animal reproduction is no different, especially when considering reproductive treatments for females. In this context, the use of mesenchymal stem cells in the treatment of ovarian insufficiency resulting from chemotherapy in rats (Feng et al., 2020; Sen Halicioglu et al., 2022) was able to increase estrogen (E2) and anti-Müllerian hormone (AMH) concentrations. Both E2 and AMH are hormones linked to ovarian activity, with E2 being secreted by follicles in advanced stages of development (pre-ovulatory follicles), while AMH is mainly associated with the recruitment of the follicular reserve. Thus, the intravenous and *in situ* application of MSCs proved effective in influencing both ends of the follicular development chain, improving the function of ovaries affected by insufficiency after chemotherapy. Additionally, pathological analysis of ovarian tissue in animals subjected to treatments showed an increase in the number of developing follicles in the ovaries of females 4 weeks after the start of treatments, confirming the beneficial effect on ovarian regulation in these cases (Feng et al., 2020).

The evaluations of the effects of MSCs on the ovary go beyond follicular count and hormonal measurements. In another study involving treatment with this cell type, the analysis of ovarian tissue after treatment with MSCs expressing genes for vascular endothelial growth factor (VEGF+MSC) demonstrated an increase in immunofluorescent expression of marked cells, especially around antral and primordial follicles, as well as the cells of the inner and outer theca and granulosa, demonstrating the activation of MSCs in these regions (Vural et al., 2019). In the same study, analyses showed an increase in the expression of TGF-ß (Transforming Growth Factor Beta 1) in tissues adjacent to the oocyte, indicating the presence of an accelerated regenerative process. All these results, along with the finding that treatment with MSCs derived from the human umbilical cord can increase the number of vessels and cords containing red blood cells in the analyzed regions of the ovaries (Yang et al., 2019), are additional positive factors for ovarian regulation.

Also acting in metabolomics, MSCs derived from the human umbilical cord were able to alter metabolism in the ovarian region. Through the activation of the PI3K pathway, treatment with this cell type increased levels of free amino acids, resulting in an increase in lipid metabolism and a decrease in monosaccharide concentration (Zhao et al., 2020), aiding in the restoration of ovarian function through another biological pathway.

## Conclusion

The treatments involving stem cells were able to act on hormonal, tissue, cellular, and metabolic levels, generating various changes that resulted in the recovery and improvement of ovarian regulation. This, in turn, enhanced key ovarian factors in animal reproduction, such as E2 and AMH levels, as well as follicular count. The successful results stem from different modes of application (*in situ* or intravenous), using cells from different sources (adipocytes, umbilical cord), and from different species (humans and rats), but ultimately reflecting the same positive outcomes regarding cell treatment related to the health and reproductive function of females. Although the MSCs therapy seems to be a great way to treatments, more studies and trials should be conducted to consolidate it and elucidate this scenario. There still is an open highway to new research in this area that, with a great quantity of studies and trials, this cell therapy would be able to contribute to the livestock efficiency, enhancing the reproduction numbers.
